# Warming Reduces Cold Hardiness of Boreal Plants but Damage Risk Varies by Species and Season

**DOI:** 10.1111/gcb.71081

**Published:** 2026-08-26

**Authors:** Francisco Campos‐Arguedas, Erica Kirchhof, Michael G. North, Kyle J. Pearson, Mark P. Guilliams, Paul J. Hanson, Al P. Kovaleski

**Affiliations:** ^1^ Department of Plant and Agroecosystem Sciences University of Wisconsin‐Madison Madison Wisconsin USA; ^2^ Environmental Sciences Division Oak Ridge National Laboratory Oak Ridge Tennessee USA

**Keywords:** boreal forest, cold hardiness risk, phenology, species‐specific dynamics, whole ecosystem warming

## Abstract

Winter warming is altering plant exposure to cold events, making it increasingly important to understand how cold hardiness dynamics respond across the dormant season. Using the Spruce and Peatland Responses Under Changing Environments (SPRUCE) experiment in northern Minnesota, we measured bud cold hardiness across four dormant seasons (2021–2025). There, a boreal peatland community was exposed to continuous active whole ecosystem warming across five levels (+0.00°C to +9.00°C) combined with atmospheric and elevated CO_2_ (+500 ppm) in open‐top chambers. Cold hardiness of two overstory ( *Larix laricina* and *Picea mariana* ) and two understory ( *Chamaedaphne calyculata* and *Rhododendron groenlandicum* ) species was evaluated semi‐regularly throughout seasons. Both understory and overstory species gained cold hardiness (acclimated) at warmer temperatures in the fall than those causing deacclimation in the spring, demonstrating different temperature responsiveness phases across the dormant season. Based on cold hardiness sensitivity, warming did not alter maximum midwinter cold hardiness (sensitivity~0°C change in cold hardiness/°C of warming) but delayed fall acclimation and, most importantly, accelerated spring deacclimation (sensitivity > 0°C). These changes resulted in increased cold damage risk in late winter and spring with warming (sensitivity > 1°C/°C) for *L. laricina* , *C. calyculata* and *R. groenlandicum* . Among overstory species, the deciduous *L. laricina* was more sensitive to warming than the evergreen *P. mariana* , indicating warming may disadvantage acquisitive species. For understory shrubs, reduced snow cover in the warmest enclosures that removed the insulating buffer and exposed buds to temperatures colder than in control plots associated with the high sensitivity of these species resulted in observed midwinter bud mortality with warming. Rather than uniformly changing cold damage risk, warming decreases risk of cold damage in fall and early winter for all species, but increases risk in late winter and spring for most species, suggesting species‐specific cold hardiness dynamics and snow‐driven microclimate may result in boreal forest composition changes.

## Graphical abstract

Whole ecosystem warming did not alter maximum midwinter cold hardiness of four boreal peatland species, but delayed fall acclimation and especially accelerated spring deacclimation. However, warming only increased cold damage risk in late winter and spring and for three of the four species, suggesting potential shifts in boreal forest composition.

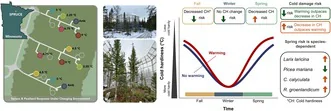

## 1 Introduction

Low temperature is a primary constraint on plant survival and distribution, particularly in temperate and boreal regions where winter conditions exert strong selective pressure on plant communities (Chapin et al. 2010; Hänninen 2016; Kellomäki et al. 2008; Neuner, Monitzer, et al. 2019; Parker 1963; Sakai and Larcher 1987a, 1987b; Stralberg et al. 2020). To persist in these environments, woody plants prepare for winter conditions by progressively increasing the cold hardiness of overwintering tissues through a dynamic physiological response that integrates daylength, and short‐ and long‐term temperature cues. Midwinter cold hardiness and deacclimation dynamics present species‐ and genotype‐specific limits that are tightly linked to the climate of origin as strongly temperature‐dependent phenotypes (Alden and Hermann 1971; Kovaleski 2022; Londo and Kovaleski 2025; Strimbeck et al. 2015; Vitasse et al. 2014). Climate change disrupts these relationships through rising temperatures and increasingly irregular seasonal temperature cycles, such as midwinter warm spells and abrupt returns of cold. These events alter cold hardiness dynamics and can leave plants vulnerable to temperatures they would otherwise tolerate depending on timing, length, and magnitude of warming exposure (Campos‐Arguedas, Kirchhof, North, Londo, et al. 2026; Ettinger et al. 2020; Wolkovich et al. 2022). Although winter warming effects on cold hardiness, dehardening, and dieback have been documented across numerous field and observational studies (Inouye 2000; Ögren 1996; Bokhorst et al. 2008; Man et al. 2016; Arora and Taulavuori 2016; Riikonen et al. 2026), experimental evidence of how climate warming will affect risk of cold damage to plants during winter at the whole ecosystem level remains limited. In particular, it is unclear whether continuous warming changes cold‐damage risk uniformly across a community and time, or instead shifts risk in species‐ or timing‐specific manners. Cold hardiness—the lowest temperature a tissue or plant can survive (i.e., greater cold hardiness means more negative values of temperature)—is not a static trait. The cold hardiness at any moment during the dormant season reflects continuous processes of acclimation and deacclimation that depend on genotypic and environmental conditions such as short‐term and long‐term thermal exposure, both of which also affect dormancy status (CaraDonna and Bain 2016; Kovaleski 2024; Neuner, Kreische, et al. 2019). As a result, a plant's cold hardiness follows a broadly predictable annual cycle of acclimation, maintenance, and deacclimation that is nonetheless sensitive to weather variability, such that vulnerability to cold damage shifts continuously through the dormant season (Campos‐Arguedas, Kirchhof, North, Londo, et al. 2026; Kovaleski 2024; Lenz et al. 2016; Neuner, Kreische, et al. 2019). This dynamic nature means that the same absolute temperature can be harmless at one point during the dormant season and lethal at another, depending entirely on the physiological state of the plant at the time of exposure.

Cold injury is often related to temperature fluctuations rather than the absolute winter minimum. Warm winter conditions can cause early deacclimation or budbreak, resulting in damage from returning deep cold or late freezes, respectively. Both instances reduce performance and survival even when long‐term thermal conditions remain within the tolerance range for a species (Campos‐Arguedas, Kirchhof, North, Londo, et al. 2026; Chamberlain et al. 2019; Lamichhane 2021; Wang et al. 2025; Weisz et al. 2011). Furthermore, species with carbon‐acquisitive strategies tend to break bud earlier to capture light and nutrients ahead of competitors, but do so at greater risk of late freeze damage, whereas species with more conservative strategies break bud later and more reliably avoid damaging freeze events, albeit at the cost of a shorter growing season (Luo et al. 2026; Maracahipes et al. 2018). The greater risk can be costly as cold injury also affects carbon acquisition by delaying canopy establishment following damage (Campos‐Arguedas, Kirchhof, North, Londo, et al. 2026; Gu et al. 2008), with effects that can span multiple seasons (Wang et al. 2025), and thus cold damage can affect climate feedback of perennial vegetation. Carbon acquisition itself can be related to cold hardiness, as nonstructural carbohydrates contribute to cold hardiness levels (Strimbeck et al. 2007; Palacio et al. 2015), which may be in turn affected by elevated atmospheric CO_2_. In some conifers, elevated CO_2_ slowed fall acclimation, reducing cold hardiness (Margolis and Vézina 1990; Guak et al. 1998), thus having the opposite effect expected based on potential increases in carbohydrate metabolism. Therefore, whether atmospheric CO_2_ alters seasonal cold hardiness dynamics in different boreal species remains unresolved.

Snow cover affects cold hardiness dynamics as a thermal buffer, insulating both aboveground bud tissue and shallow roots from extreme temperature fluctuations, such that plant tissues beneath snow experience a narrower range of temperatures than those above snow (Schaberg et al. 2011). While this buffering effect is relevant across perennial species, understory plants are particularly vulnerable when snow is absent or lost early due to warming, as the entire plants (roots and canopy) become exposed to a wider range of temperature fluctuations (Kreyling et al. 2012; Schaberg et al. 2011). Adaptive cold hardiness regulation is therefore critical not only for preventing episodic tissue damage, which is governed by within‐year temperature regimes and microclimate conditions, but also for determining long‐term survival and adaptation of woody perennial plants, and their contribution to the carbon cycle.

Here, we assessed seasonal cold hardiness responses over four winter seasons (2021–2025) at the SPRUCE (Spruce and Peatland Responses Under Changing Environments) experiment in northern Minnesota (https://mnspruce.ornl.gov). At SPRUCE, a boreal peatland community is exposed to continuous warming across five target levels (+0.00°C, +2.25°C, +4.50°C, +6.75°C, and + 9.00°C above ambient temperature) using open‐top chambers, at either atmospheric or elevated CO_2_ (+500 ppm) (Hanson et al. 2017). We examined temporal changes in bud cold hardiness as a response to the different environmental conditions for four co‐occurring species from two functional groups: a deciduous conifer (larch, *Larix laricina* (Du Roi) K. Koch) and an evergreen conifer (black spruce, *Picea mariana* (Mill.) B.S.P.) make up the overstory, and two evergreen shrubs (leatherleaf, *Chamaedaphne calyculata* (L.) Moench; and Labrador tea, *Rhododendron groenlandicum* (Oeder) Kron & Judd) represent understory species. We quantified the effects of warming and CO_2_ enrichment on seasonal cold hardiness at two levels: across contrasting functional groups (overstory and understory), and between species within the same functional group but different foliage habits (deciduous and evergreen). Together, these comparisons allow us to assess how climate change can reshape the thermal limits of winter survival across a boreal plant community. Using an expansive cold hardiness dataset collected at SPRUCE, we tested the following hypotheses: (H1) whole ecosystem warming reduces bud cold hardiness; (H2) CO_2_ enrichment increases cold hardiness due to enhanced metabolism; (H3) reduced cold hardiness results in increased cold damage risk; and (H4) cold hardiness and damage risk is species‐specific, varying with functional group and foliage habit.

## 2 Materials and Methods

### 2.1 Site Description and Experimental Design

**FIGURE 1:**
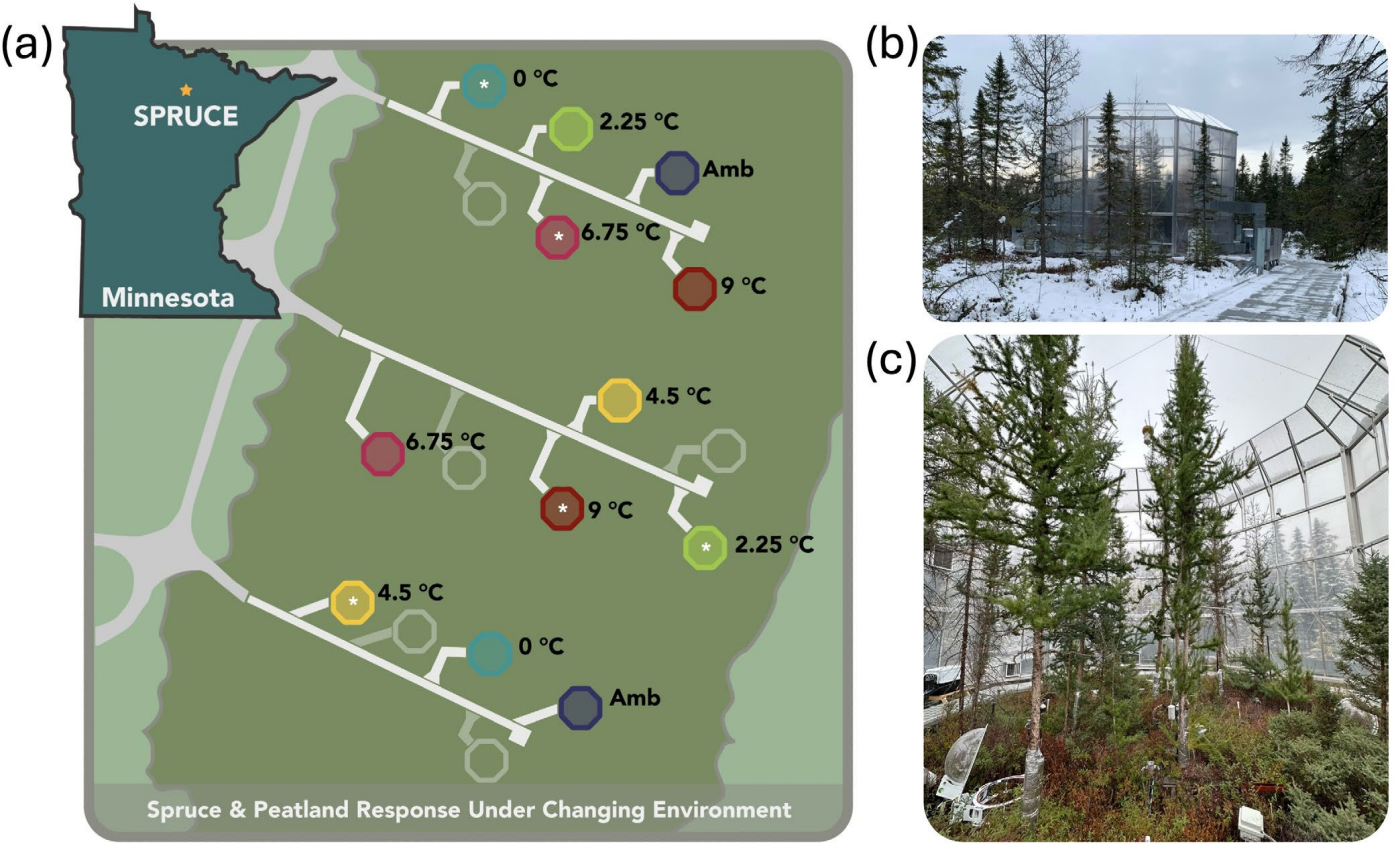
Layout of the SPRUCE experimental enclosures and associated treatments. (a) Location of the SPRUCE experimental site within the Marcell Experimental Forest in northern Minnesota, and schematic of the arrangement of enclosures and their assigned warming treatments, ranging from +0.00°C to +9.00°C above ambient. Plots with an asterisk (*) were enriched with CO_2_ at ~500 ppm above atmospheric. (b) Exterior view of an experimental enclosure. (c) Interior view of an experimental enclosure. Map lines delineate study areas and do not necessarily depict accepted national boundaries.

Plant material was collected from trees and shrubs within the SPRUCE (Spruce and Peatland Responses Under Changing Environments) experiment. SPRUCE is located at the Marcell Experimental Forest in Minnesota, USA, at the S1‐Bog, in the southern edge of the boreal zone (47°30.476′ N; 93°27.162′ W) (Figure 1a). The S1‐Bog is an ombrotrophic peatland with large and rapid diurnal and seasonal temperature fluctuations (subhumid continental climate) (Verry et al. 1988). The mean annual temperature, based on a 48‐year average (1961–2009) is 3.4°C, with recorded air temperatures ranging from −45°C to 38°C throughout the year (Sebestyen et al. 2011). Canopy vegetation is dominated by the tree species black spruce [ *Picea Mariana* (Mill.) B.S.P] and American larch [ *Larix laricina* (Du Roi) K. Koch]. The understory includes ericaceous shrubs, from which the dominant two species were included in collections: Labrador tea [ *Rhododendron groenlandicum* (Oeder) Kron & Judd], and leatherleaf [ *Chamaedaphne calyculata* (L.) Moench.] (Hanson et al. 2017).

Climate conditions were manipulated within 10 large open‐top enclosures (12.8 m diameter, 7 m height) built surrounding plants in 2015 (Figure 1b,c). Each enclosure was constructed with double‐walled transparent greenhouse panels to allow increase in both air and soil temperature, and in CO_2_ concentration. Additionally, a subsurface corral provides hydrologic isolation, allowing the soil to be warmed to match levels of air temperature warming (Hanson et al. 2017).

Five levels of continuous active ecosystem warming were applied, with target temperature treatment increases of: +0.00°C, +2.25°C, +4.50°C, +6.75°C, and +9.00°C relative to the non‐heated control plot (+0.00°C; plot 6), against which the SPRUCE warming treatments were regulated. Temperatures were also monitored in two adjacent non‐enclosed plots, here referred to as “Amb” or “Ambient,” which provided an external, non‐enclosed reference. The evenly spaced levels follow a regression‐based design to derive continuous temperature‐response functions rather than reproducing specific climate projections (Cottingham et al. 2005; Kardol et al. 2012; Hanson et al. 2017). The highest level (+9.00°C) was set beyond near‐term projections to define the upper bound of the response gradient (Cavaleri et al. 2015; Kayler et al. 2015; Torn et al. 2015).

Temperatures were recorded at various aboveground heights and belowground depths at 30‐min intervals across each plot. For our analyses, we used air temperature measured at 2 m height for overstory species and temperature at the soil surface (0 m) for understory shrubs. Two enclosures were assigned to each warming level, with one enclosure per level also receiving elevated CO_2_ (~500 ppm above atmospheric). The SPRUCE project operated year‐round with deep‐soil heating (−2 m) initiated in June 2014, air warming beginning in August 2015 (Hanson et al. 2016), both to the same degree, and CO_2_ fumigation starting in June 2016 (Hanson et al. 2021). The enclosures were constantly heated using propane‐fired heat exchangers and a system of air blowers and conduits; the reference for both air and belowground heating was the non‐heated control plot (+0.00°C; plot 6). A more detailed description of the experimental setup and treatments is provided within (Hanson et al. 2017).

### 2.2 Cold Hardiness Determination

Bud cold hardiness was monitored across four seasons (2021–2025) for all four species using differential thermal analysis (DTA) as described by (Mills et al. 2006). Briefly, buds were excised from the twigs and shoots avoiding damage to their internal structure and placed on thermoelectric modules (TEMs). Samples were then exposed to decreasing temperature in an environmental chamber. The temperature program held temperature at −5°C for 5 h, followed by a decrease to −55°C at a rate of −6°C h^−1^, and a final hold at −55°C for 2 h. During this process, water within bud tissue supercools until the cold hardiness level of the structure. Once intracellular water freezes, heat is released, which can be detected as a low temperature exotherm (LTE) that represents the killing temperature (Quamme 1991). LTEs were recorded using to a Keithley 6510 multimeter data acquisition system (Keithley Instruments), with LTE signals referenced to temperatures recorded by a thermocouple (22 AWG). DTA is an appropriate technique for species that use supercooling in their buds, which includes those studied here (Sakai and Larcher 1987a), and cold hardiness measurements were supported through a comparison to the visual browning method for cold hardiness determination (Figure S1; Villouta et al. 2020).

Samples were collected at semi‐regular intervals from August through May of each of the four seasons. Buds were placed in plastic bags with moist tissue paper, transported in a cooler with ice and water mix (0°C) and stored at 4°C until analysis. Occasionally, buds were shipped overnight in a cooler with ice packs, which does not significantly affect cold hardiness measurements (Londo et al. 2023). To minimize destructive sampling within plots, bud samples were pooled from multiple individuals of the same species within each enclosure. Typically, 10–15 buds were used to assess cold hardiness per species per enclosure (~20,500 total buds for cold hardiness measurements over four years for all four species). Outliers were identified and removed using studentized residuals exceeding ±2.95 prior to analyses.

### 2.3 Temperature Characterization

Temperature effects on cold hardiness were examined at two temporal scales: inter‐collection responses capturing the immediate effect of thermal conditions between consecutive sample collection dates (short‐term), and responses reflecting the cumulative intra‐seasonal thermal environment experienced by each plot from midsummer until the time of each sample collection within a season (medium‐term).

#### 2.3.1 Inter‐Collection Temperature Effects

To characterize the thermal conditions experienced between consecutive sample collections, we calculated the mean air temperature over the inter‐collection interval preceding each sampling date. For each treatment plot and season, the inter‐collection (ic) mean temperature ${\bar{T}}_{\mathrm{ic}}$ for collection date c was computed as:

$${\bar{T}}_{{\mathrm{ic}}_{c}}=\frac{1}{n_{\mathrm{ic}}}\sum_{d=c-1}^{c}T_{d}$$

where $T_{d}$ is the daily mean temperature on day d within the interval between collection date c−1 and collection date c, and $n_{\mathrm{ic}}$ is the number of days in that inter‐collection interval. This variable captures the thermal conditions most immediately preceding each cold hardiness measurement and was used to assess short‐term responsiveness of cold hardiness to recent temperature in absolute values.

#### 2.3.2 Cumulative Intra‐Seasonal Temperature Effects

To characterize the cumulative thermal environment of each plot across the season, we computed the average relative temperature adjustment (effective ∆ *T*) for each plot in relation to ambient temperature plots. For each collection date c, the cumulative mean temperature for each plot p $\left({\bar{T}}_{p,c}\right)$ was calculated as the mean of all daily temperatures from 1 August of through each collection date c within a season:

$${\bar{T}}_{p,c}=\frac{1}{n_{c}}\sum_{d=d_{0}}^{c}T_{p,d}$$

where $d_{0}$ is 1 August and $n_{c}$ is the number of days from $d_{0}$ to collection date c. The reference temperature for each collection was defined as the mean of ${\bar{T}}_{p,c}$ across the two ambient reference plots (both non‐warmed and without enclosures). The effective temperature differential between any plot and ambient plot temperatures (∆ *T*) was then calculated as:

$$\Delta T_{p,c}={\bar{T}}_{p,c}-{\bar{T}}_{\mathrm{ref},c}$$

where ${\bar{T}}_{\mathrm{ref},c}$ is the cumulative mean temperature of the reference plots for each collection within a season. Thus, ∆ *T* represents how much warmer each plot was relative to ambient conditions up to each sampling point and was used as the predictor of cumulative intra‐seasonal warming effects on cold hardiness.

### 2.4 Data Analysis and Statistical Tests

All data were analyzed using R (ver. 4.4.0; R Core Team 2024) within R Studio (Posit team 2024). Given the regression‐based experimental design, warming treatment was treated as a continuous variable throughout, represented by ΔT or ${\bar{T}}_{\mathrm{ic}}$, and only first‐degree polynomial regressions were considered.

#### 2.4.1 CO_2_ and Treatment Effects

A full model was fit for cold hardiness using CO_2_ concentration, plot temperature ΔT, and collection as explanatory variables. Cold hardiness was not significantly affected by elevated CO_2_ or its interaction with warming treatment across species (Notes S1, Table S1), and CO_2_ was therefore excluded from all subsequent analyses.

#### 2.4.2 Short‐Term Thermal Responsiveness

To quantify short‐term sensitivity of cold hardiness to recent temperature conditions, we calculated the change in cold hardiness between consecutive sampling dates as follows:

$$\Delta {\mathrm{CH}}_{c}={\mathrm{CH}}_{c}-{\mathrm{CH}}_{c-1}$$

where ${\mathrm{CH}}_{c}$ is the mean cold hardiness on collection date *c* and ${\mathrm{CH}}_{c-1}$ is the mean cold hardiness on the previous sampling date, calculated separately for each species, treatment, and season. ΔCH values were then matched to their corresponding inter‐collection mean temperatures ${\bar{T}}_{\mathrm{ic}}$ and each observation was assigned to its respective seasonal phase. To characterize phase‐specific thermal responsiveness, phase centroids were calculated as the mean inter‐collection temperature and mean ΔCH for all observations within each phase and functional group. Bivariate 95% confidence ellipses were fitted assuming a normal distribution using stat_ellipse() in ggplot2 (Wickham 2016) to visualize the dispersion of observations around each centroid and assess overlap between phases.

#### 2.4.3 Cumulative Intra‐Seasonal Warming Sensitivity

To quantify the sensitivity of cold hardiness to cumulative intra‐seasonal warming at each collection, separate linear models were fitted for each species and collection using Δ*T* as the sole predictor:

$$CH_{c}=\beta_{0}+\beta_{1}\Delta T_{c}+\varepsilon_{i}$$

where $CH_{c}$ is cold hardiness for collection c, $\beta_{0}$ is the intercept representing cold hardiness at approximately ambient conditions (Δ*T* = 0°C), $\beta_{1}$ is the slope associated with the cold hardiness sensitivity to warming (°C cold hardiness change per °C of warming), and $\varepsilon_{i}$ is the residual error. Slopes were estimated only for collection periods with at least four unique target temperature levels represented to ensure stable parameter estimation. Slopes were estimated separately for each collection to capture phase‐specific variation in warming sensitivity across the season. The warming effect was considered significant when the sensitivity slope (β₁) differed from zero (*p* < 0.05), based on the *t*‐test of the slope coefficient (*n* = 10 enclosures; df = 8). Significance therefore reflects the continuous temperature response across the gradient rather than pairwise contrasts between treatment levels.

Within each species and season, collection‐specific slopes (β₁) were compared pairwise using estimated marginal trends (emmeans package; Lenth 2023) with Tukey adjustment (α = 0.05). To account for seasonal variation in the magnitude of both inter‐collection and cumulative intra‐seasonal temperature responses, data were grouped into three phases based on overall cold hardiness patterns observed across species and years for interpretation: acclimation (1 Aug—31 Oct), maintenance (1 Nov—28/29 Feb), and deacclimation (1 Mar—budbreak).

## 3 Results

### 3.1 Warming Results in Uneven Effects Depending on Plant Functional Group

**FIGURE 2:**
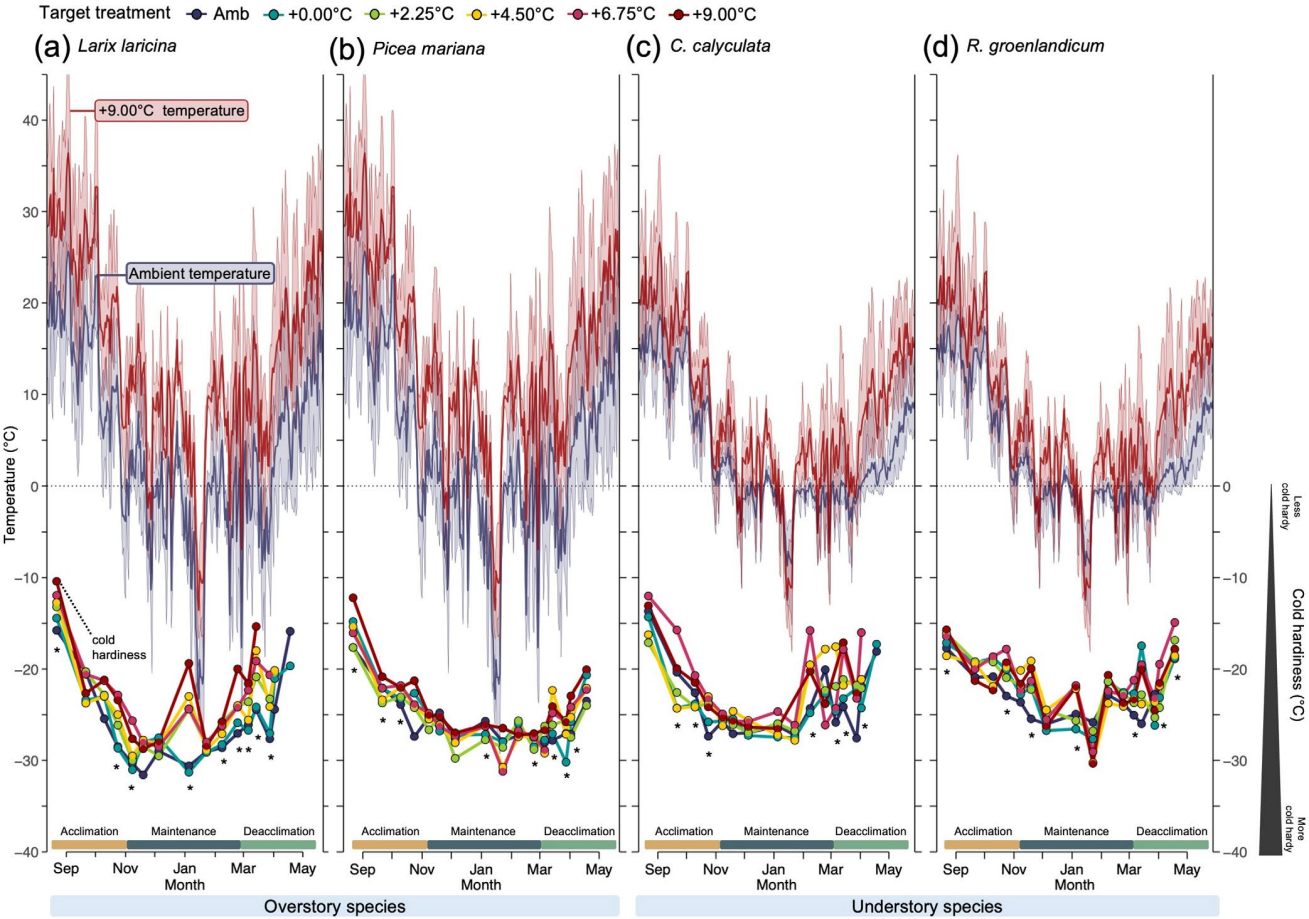
Seasonal patterns of bud cold hardiness under different warming regimes. Mean bud cold hardiness trajectories and temperature conditions during the 2023–2024 season for (a) *Larix laricina* , (b) *Picea mariana* , (c) *Chamaedaphne calyculata* , and (d) *Rhododendron groenlandicum* . Mean daily temperatures (solid lines) and minimum and maximum temperatures (ribbons) are shown for the most extreme conditions: Ambient and +9.00°C target warming. Temperature data for *Larix laricina* and *Picea mariana* reflect 2 m temperature measurements, while temperatures for *Chamaedaphne calyculata* and *Rhododendron groenlandicum* reflect surface‐level measurements capturing the near‐ground microclimate relevant to understory shrubs. Asterisks (*) indicate collections where cold hardiness is significantly affected by warming based on linear regression to ∆ *T* (*p* < 0.05). Each cold hardiness value (points) for each species, temperature treatment, and time is the average of measurements in two plots (*n* = 2). The exact number of buds used for the average are provided in Table S2 by collection, species, and year. Seasonal trajectories for all four seasons are shown in Figure S4. Comparisons between ambient (non‐enclosure) and warming treatments are shown for *Larix laricina* in Figure S5.

Target warming treatments at the SPRUCE experiment are calibrated based on 2 m air temperature measurements taken on a non‐heated control plot (+0.00°C) (Hanson et al. 2017), resulting in evenly distributed temperature differentials across treatments at tree canopy height (Figure 2a,b, Figure S2a). However, conditions were considerably more variable during dormant seasons at the level of understory species due to changes in snow cover dynamics across the season (Figure 2c,d, Figure S2b). Snow cover acted as a long‐lasting insulating layer that buffered surface temperatures in colder treatments when compared to 2 m temperature. However, in more highly warmed plots, lack of snow cover for understory plants resulted in exposure to a wider range of temperatures, with more negative temperatures during cold events and higher temperatures during warm events, compared to snow‐covered plants in less warmed plots (Figures S3 and S4). Due to the snow cover dynamics, thermal regimes differed considerably between overstory and understory species. Therefore, all analysis used temperatures recorded at the relevant height for each functional group (2 m for overstory and surface level for understory), rather than the target warming treatments assigned to each enclosure.

Seasonal cold hardiness patterns were consistent across years in all four species (Figure 2, Figure S4). Overstory and understory species gained cold hardiness with decreasing temperatures in the fall, but with apparent delays due to the warming treatments (Figure S5). In midwinter, there are generally no significant differences across treatments, with exception of periods of considerable warm weather. Warming advanced loss of cold hardiness in the spring, particularly in overstory species. More erratic cold hardiness responses were observed in understory species, where warming caused snow cover loss that resulted in warmer treatments experiencing lower temperatures than ambient (e.g., temperatures in January 2024—Figure 2c,d). Elevated CO_2_ had no significant effect on cold hardiness (Notes S1), and therefore we explore here only the effect of temperature. Overall, cold hardiness presents three general phases in which we explored inter‐collection and cumulative intra‐seasonal thermal responsiveness: acclimation (1 August—31 October), maintenance (1 November—28/29 February), and deacclimation (1 March—budbreak).

### 3.2 Short‐Term Thermal Responsiveness Is Phase Dependent

**FIGURE 3:**
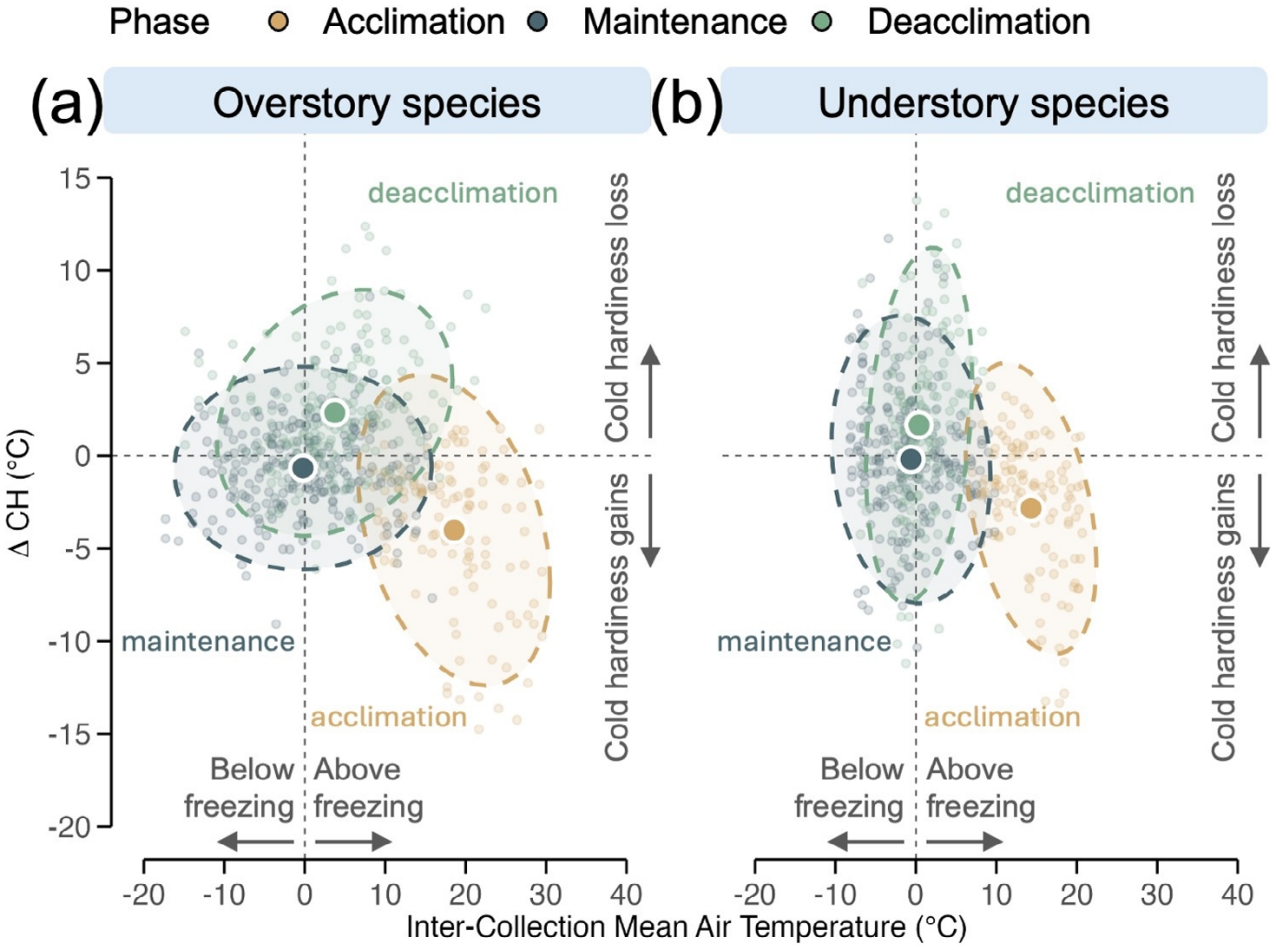
Short‐term thermal responsiveness of cold hardiness across physiological phases. Relationships between inter‐collection mean air temperature and short‐term changes in bud cold hardiness (∆CH) for (a) overstory (b) and understory species. Negative ∆CH values indicate hardiness gain (acclimation) and positive values indicate hardiness loss (deacclimation) between consecutive sample collection points. Inter‐collection mean air temperature is the average temperature between consecutive sample collection points. Points represent individual observations during acclimation (golden), midwinter maintenance (dark teal), and deacclimation (sage green) phases; filled circles indicate phase centroids and dashed ellipses represent 95% confidence regions for each phase. Treatment‐specific responses are shown in Figure S6.

Short‐term thermal responsiveness varied across physiological phases (Figure 3). We characterized the distribution of observations within each phase using phase‐specific centroids and bivariate 95% confidence ellipses. Each phase occupied a distinct region of the thermal and cold hardiness space (i.e., different boundaries of ellipses in the horizontal and vertical axis, respectively), with broadly consistent patterns in transition through phases across overstory and understory species.

During acclimation, buds consistently gained cold hardiness (negative ∆CH) despite experiencing warmer inter‐collection temperatures than during other phases (Figure 3). This reflects the late summer and early fall thermal environment under which most cold hardiness acquisition occurs (Figure 2, Figure S4). During midwinter maintenance, ∆CH values clustered near zero across a wide temperature range. This indicates that once buds reached their approximate maximum hardiness, short‐term temperature fluctuations have little influence on hardiness status—a pattern consistent across both functional groups. During deacclimation, buds lost cold hardiness with rising spring temperatures. Warmer treatments amplified this loss more than they affected fall acclimation or midwinter maintenance (Figure S5), demonstrating that spring warming has a disproportionate effect on cold hardiness dynamics over an annual cycle. Notably, cold hardiness loss during deacclimation occurred at lower temperatures than those driving hardiness gain during acclimation, reflecting a fundamental asymmetry in the thermal control of seasonal hardiness dynamics (Figure 3). In particular, understory species showed a greater range of ∆CH within a narrower range of above‐freezing inter‐collection temperatures during deacclimation, showing the higher responsiveness of cold hardiness trajectories to temperature compared to overstory species.

### 3.3 Seasonal Warming Sensitivity Suggests Temporally‐ and Species‐Specific Risk

**FIGURE 4:**
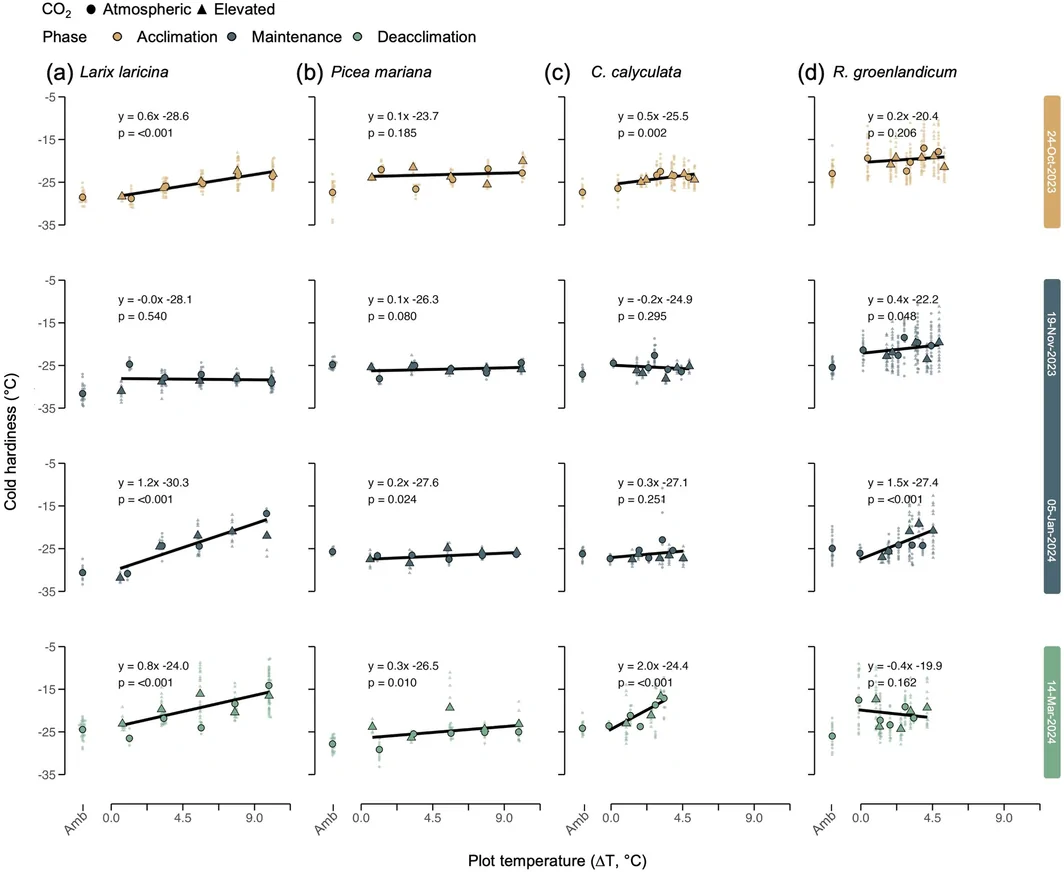
Phase‐specific cold hardiness responses to warming. Cold hardiness measured at each warming level during the 2023–2024 season for (a) *Larix laricina* , (b) *Picea mariana* , (c) *Chamaedaphne calyculata* and (d) *Rhododendron groenlandicum* , organized by seasonal phase: Acclimation (golden), maintenance (dark teal), and deacclimation (sage green). Small points show single bud measurements, while larger points show averages within a plot for each collection date. Solid lines represent linear regression fits. Ambient plots are the reference for temperature (i.e., a ∆ *T* of 0 means temperature equal to ambient plots). Full seasonal trajectories for all species and years are shown in Figure S4; additional treatment‐ and collection‐level patterns are shown in Figures S11–S14.

Warming sensitivity, expressed as the slope of cold hardiness change per degree of cumulative intra‐seasonal warming (∆*T*) for each collection, revealed clear differences between plant functional groups and phase structure across years (Figure 4; Table S3). Under these conditions, sensitivity exceeding 1°C per °C of warming indicates that cold hardiness loss outpaces the change in temperature by warming treatments, thus elevating cold damage risk by effectively reducing safety margins, whereas sensitivities below 1 mean lower cold damage risk with warming.

The cumulative effect of temperature differed for the two functional groups. For overstory species, warming treatments produced relatively even warming throughout the dormant season, with ∆ T minimum and maximum ranges of 8.54°C to 9.25°C between the +0.00°C and +9.00°C treatments (i.e., distribution of points along the *x*‐axis within Figure 4a,b, *y*‐axis within Figure S7). For understory species, however, snow cover fluctuations resulted in more variable conditions, including temperature inversions related to target treatments. This resulted in a compressing of the effective ∆ *T* range over time (2.02°C to 4.50°C; Figure 4c,d, Figure S7).

The calculation of sensitivity is exemplified for four timepoints (Figure 4). The effect of ∆ T on cold hardiness is significant during acclimation but shifts to nonsignificant during the maintenance period. An exceptionally warm period in late December 2023 and early January 2024 (with temperatures ~7.0°C above seasonal norms for December (Figure S8) and largely above freezing for 17 days in the warmest plot) triggered transient midwinter deacclimation in warmed plots, leading to high sensitivity in all species with the exception of leatherleaf (Figure 4). During spring deacclimation, warming sensitivity returned to significant levels.

**FIGURE 5:**
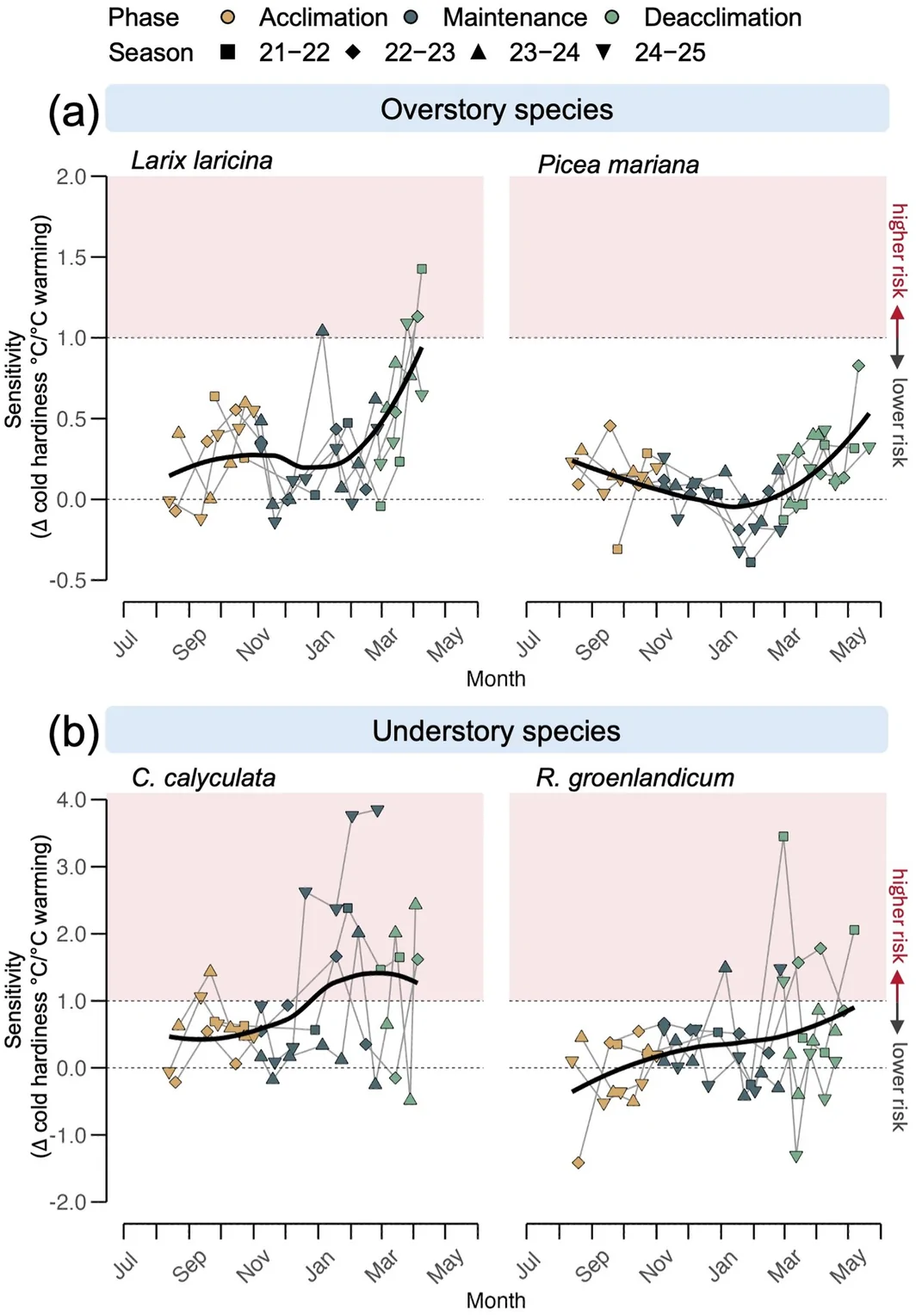
Warming sensitivity of bud cold hardiness over the dormant season. Slopes of bud cold hardiness in response to cumulative warming changes in temperature experienced by plants for (a) overstory and (b) understory species across acclimation (golden), maintenance (dark teal), and deacclimation (sage green) phases. Each point represents the slope of cold hardiness change per °C warming using ∆ *T* for an individual collection across four seasons. Thick black lines show the loess‐smoothed trend; thin lines connect points within individual seasons. A sensitivity > 1°C/°C indicates higher cold damage risk or reduced safety margins, whereas sensitivity < 1°C/°C indicates lower risk. A sensitivity = 0°C/°C means no effect of temperature. Only slopes with *n* ≥ 4 treatments were retained for overstory and understory species.

When explored over time, all four species show similarities in the trajectories of sensitivity (Figure 5). In August, sensitivity is near zero as buds have only their inherent levels of cold hardiness as they are formed regardless of summer temperatures. Slight increases in sensitivity are observed during acclimation and reduced during maintenance as plants reach their maximum cold hardiness. All species show strong increases in sensitivity during the deacclimation phase. These seasonal sensitivity patterns were similar when inter‐collection mean temperature (our measurement of short‐term temperature responses) is used as the predictor instead of ∆ *T* (Figure S9), suggesting that the observed dynamics are robust and not dependent on the temperature metric used.

Among overstory species, larch was more sensitive to warming than black spruce across all phases (Figure 5a). At the end of an unusual midwinter warm spell spanning late 2023 and early 2024, larch showed a sensitivity of 1.2°C per °C on 5 January 2024 (Figure 4a), while black spruce exhibited a weaker response (0.2°C per °C; Figure 4b). Both species fully reacclimated when cold temperatures returned. Importantly, at the end of the deacclimation period, larch showed sensitivity levels closer to and above 1.0°C per °C, whereas black spruce still showed comparatively more conservative responses even during this most responsive phase (~0.5°C per °C).

Understory species showed similarly phase‐dependent responses (Figures 4c,d and 5b), but their magnitude was strongly modulated by warming‐induced reductions in snow cover. This resulted in sensitivities higher than 1°C per °C occurring earlier in the season and to much greater magnitudes in both directions compared to overstory species. Understory species also showed higher year‐to‐year variation that reflects different snow conditions across years.

Changes in cold damage risk were not uniform across the dormant season. While cold hardiness was most often significantly affected by treatments during acclimation (sensitivity > 0°C per °C), cold damage risk was always reduced with increasing temperatures in this phase (sensitivity ≤ 1°C per °C). During the maintenance period, risk was substantially reduced with warming for most species. Higher risk of bud cold damage with warming (sensitivity > 1°C per °C) was observed in larch and understory species with progression of the dormant season, but not in black spruce. However, for the three species that crossed that threshold, the time when it occurred within the season varied. Highest risk was observed in understory species, amplified by snow cover loss that disproportionately increased their exposure to temperature extremes, resulting in observable bud damage in warmer plots (Figure S10).

## 4 Discussion

Here we examined the effects of experimental whole ecosystem warming on cold hardiness dynamics in boreal woody perennial plant species. Our results demonstrate that climate warming alters cold hardiness dynamics in both overstory and understory species, but the magnitude and consequences of these responses vary between seasonal phases and species. Despite widespread warming across northern forests over the past century (Contosta et al. 2019; Sebestyen et al. 2011), we found no evidence that warming alters maximum midwinter cold tolerance—even at the warm range edge of the boreal forest where this experiment is located. Instead, warming delayed fall acclimation and most importantly accelerated spring deacclimation, particularly increasing plant vulnerability during the spring transitional period when cold hardiness declines faster than environmental cold risk recedes. These phase‐ and species‐specific responses reduce safety margins most strongly during transitional seasons and suggest that climate‐driven shifts in seasonal timing may increasingly influence cold damage risk and future species composition in boreal forests.

### 4.1 Temperature Dictates Climate Change Effects on Phase‐Specific Plant Vulnerability

The experimental design for SPRUCE does not allow for pairwise comparisons of treatments, and effects of temperature and CO_2_ were evaluated using linear regressions—which appropriately fit the cold hardiness responses observed (e.g., Figure 4). Elevated CO_2_ did not influence cold hardiness dynamics across species or phases. Cold hardiness is related to carbohydrate concentrations, including the cold hardiness of buds as they form in the summer (Morin et al. 2007). CO_2_ enrichment has been shown to not increase concentration of nonstructural carbohydrates in tissues, even when total growth increases (Tognetti and Johnson 1999; Bigras and Bertrand 2006). Our results suggest that current CO_2_ levels pose no direct limitation on carbon metabolism related to cold hardiness—even at higher temperatures where respiration can be higher (Velappan et al. 2022). Though rising atmospheric CO_2_ concentration will not directly alter the freeze risk of perennial plants, its effect in natural ecosystems will be a result of its greenhouse gas properties increasing temperature.

Incremental warming delayed fall acclimation and accelerated spring deacclimation, together extending the period during which tissues were most susceptible to damaging temperatures at both ends of the dormant season (Figure 2). The delay in fall acclimation reflects a temperature‐driven shift in the timing of cold hardiness acquisition rather than a reduction in the inherent capacity of boreal species to withstand extreme winter temperatures (Chapin et al. 2010; Neuner 2014; Stralberg et al. 2020). Cold hardiness gains still occur at a fast pace despite warm temperatures (Figure 3), leaving wide safety margins during fall. In spring, warming sensitivity was strongest, substantially narrowing the safety margin against late frost damage for larch and understory species. This phenological mismatch where the cold hardiness loss outpaces the seasonal increase in temperature is increasingly recognized as a key driver of cold damage in forests and woody perennial crops (Bigler and Bugmann 2018; Chamberlain and Wolkovich 2021; Richardson et al. 2018).

The exceptional warm period in December 2023 and January 2024 illustrates that increased cold damage risk derived from warming is not restricted to the spring deacclimation phase. Warming events that are intense and long enough, particularly occurring late during winter or early spring, can cause deacclimation that can be detrimental if reacclimation does not occur fast enough to avoid damage from returning intense cold (Kovaleski 2024), which here we observe as highly species‐specific effects. Rather than altering the maximum depth of midwinter hardiness even at the warm range edge of the boreal forest, warming effects were expressed almost exclusively during seasons when plants were actively gaining or losing cold hardiness, highlighting that studies using only midwinter measurements may substantially underestimate climate‐driven shifts in vulnerability.

Seasonal sensitivity shifts of cold hardiness responses reflect the physiological regulation of dormancy. During winter maintenance, buds remain dormant and exhibit limited responsiveness to temperature variation (Figures 4 and 5), consistent with physiological constraints that stabilize cold hardiness against short‐term fluctuations (Lenz et al. 2016; Vitasse et al. 2014). In contrast, plants become more temperature‐responsive with dormancy progression (Kovaleski 2022, 2024; North et al. 2024; North and Kovaleski 2024) and even brief warm periods during these phases can rapidly accelerate cold hardiness loss while damaging freeze events remain likely. Short‐term temperature analyses confirmed this mechanism directly, showing that cold hardiness responsiveness was minimal during the maintenance phase, but higher during acclimation and deacclimation. Particularly, the high positive ∆CH even at low above‐freezing temperatures during the deacclimation phase identifies late winter and spring as the period of greatest warming‐driven freeze risk (Figure 3).

### 4.2 Warming Sensitivity Is Related to Species Habits and Functional Types

Species differed strongly in their sensitivity to warming during transitional seasons, reflecting contrasting life history strategies along the carbon acquisitive‐conservative axis. As a deciduous conifer, larch exhibited rapid loss of cold hardiness as temperatures increased in spring (Figure 2) and showed higher sensitivity during transitional phases compared to black spruce (Figure 5). Higher sensitivity may be the result of an acquisitive strategy in which early phenological responsiveness maximizes the potential growing season but increases exposure to episodic cold events during transitions as cold hardiness loss in deciduous species precedes budbreak and the onset of photosynthesis (Bowling et al. 2024; Givnish 2002; Vitasse et al. 2014). Black spruce maintained slower and more conservative responses across all phases. As an evergreen conifer, black spruce retains foliage and the capacity to resume photosynthesis soon after warm spring temperatures return—in fact, showing higher sensitivity for spring “green‐up” than larch (Richardson et al. 2018), without requiring rapid bud deacclimation and budbreak to secure carbon gain (Dusenge et al. 2023; Givnish 2002; Öquist and Huner 2003). The more rapid foliage green‐up with warming in black spruce resulted in frost damage to foliage in April 2016 at SPRUCE, but not to buds (Richardson et al. 2018), suggesting different cold hardiness dynamics may exist for foliage and buds in evergreen species. These contrasting strategies in carbon acquisition for deciduous and evergreen species produced substantially different bud cold hardiness safety margins under warming. Therefore, both warming and greater temperature variability resulting from climate change may increasingly disadvantage the acquisitive species with higher cold hardiness sensitivity relative to more conservative counterparts, resulting in overstory composition shifts due to cold damage.

Warming also altered cold vulnerability through indirect microclimatic pathways that disproportionately affected understory species. Reduced snow cover in warmer enclosures eliminated the insulating buffer that normally moderates near‐surface winter temperatures which affect cold hardiness (Bokhorst et al. 2009; Richardson et al. 2013, 2018; Palacio et al. 2015). Paradoxically, this exposed understory buds in the plots with the highest warming treatments to temperatures colder than within ambient and least warmed plots (Figure S2; Riikonen et al. 2026), such that measured microclimate temperatures were not proportional to the intended warming treatment. This temperature reversal likely explains the nonsignificant and occasionally negative sensitivities observed during spring deacclimation, underscoring that surface‐level microclimate and cold hardiness dynamics cannot be reliably inferred from typical weather station height air temperature in ecosystems where snow dynamics are changing (De Frenne et al. 2013; Gates 1912; Kreyling et al. 2012; Neuner et al. 1999). Reduced snow cover observed in warmer plots in the SPRUCE experimental system may partially result from the methods used for warming and should be viewed as one plausible pattern with future real‐world warming. That is, future snow depths in nonexperimental systems may vary from those available for study in SPRUCE depending on changes in precipitation patterns, wind dynamics, and other factors not captured by the experiment.

The greater range of ∆CH in smaller range of temperatures observed in understory species during deacclimation (Figure 3b) likely reflects their dependence on snow cover as a thermal buffer that typically moderates spring temperature exposure in boreal understories (Charra‐Vaskou et al. 2026; Steger et al. 2013). The distinct warming sensitivities observed between the two understory species, despite identical enclosure conditions, further suggest that species‐specific tolerance to microclimatic variability, rather than mean warming alone, will determine understory compositional change under future climate scenarios (De Frenne et al. 2013; Marty et al. 2017; Panchard et al. 2023; Steger et al. 2013; Zellweger et al. 2019). Critically, bud damage was observed in both understory species in the warming treatments—particularly +6.75°C and +9.00°C—during two of the four study seasons: driven by a warm spell in December 2023 and early January 2024 during the 2023–2024 season, and by warming‐induced snow cover loss during the 2024–2025 season (Figure S10). This is consistent with longstanding experimental evidence that reduced snow insulation exposes understory plants to damaging freeze events (Gates 1912; Kreyling et al. 2012; Neuner et al. 1999). Here we provide experimental evidence that near‐term air warming will cause cold injury through midwinter snowmelt in boreal plant communities.

## 5 Conclusion

Together, our results demonstrate that cold hardiness is a dynamic, phase‐related trait, and that species‐specific representation of these dynamics, which is limited in the literature (Jones et al. 2024; Kovaleski 2022; Wolkovich et al. 2012, 2014), is essential for predicting forest resilience, productivity, and climate feedbacks under warming. Studies of species distribution or productivity that assume constant cold hardiness or neglect phase‐specific sensitivity risk may thus systematically under or overestimate climate‐driven damage as winter and spring temperature variability intensifies, depending on when sensitivity is evaluated (Kovaleski 2024; Neuner 2014; Richardson et al. 2018). Although warming may lengthen growing seasons for some species, increased exposure to episodic cold events during transitional seasons, especially in spring and amplified by snow cover loss in understory microclimates, may offset these gains through tissue damage or shifts in competitive dynamics. For species of low stature, snow loss under warming was associated with bud mortality in understory shrubs, highlighting snow cover as a main factor of winter survival. Differences in seasonal cold hardiness responses among species with contrasting carbon acquisition strategies may alter competitive advantages and facilitate range shifts under continued climate warming, particularly among species with more acquisitive growth habits (Lenz et al. 2013; Londo and Kovaleski 2025; Savage and Cavender‐Bares 2013). Integrating temporally determined vulnerability, microclimatic heterogeneity, and snow‐mediated insulation into predictive models will therefore be critical for accurately projecting future changes in carbon sequestration, biodiversity, and ecosystem services across temperate and boreal forests.

## Author Contributions

**Mark P. Guilliams:** resources, writing – review and editing. **Francisco Campos‐Arguedas:** data curation, investigation, formal analysis, visualization, writing – original draft, resources. **Paul J. Hanson:** conceptualization, funding acquisition, writing – review and editing. **Michael G. North:** investigation, resources, writing – review and editing. **Erica Kirchhof:** investigation, resources, writing – review and editing. **Al P. Kovaleski:** conceptualization, data curation, formal analysis, funding acquisition, investigation, resources, visualization, writing – review and editing, supervision. **Kyle J. Pearson:** resources, writing – review and editing.

## Funding

National Institute of Food and Agriculture, United States Department of Agriculture, McIntire Stennis projects 7002619 and 1027327 (APK). United States Department of Energy, Office of Science, Office of Biological and Environmental Research.

## Conflicts of Interest

The authors declare no conflicts of interest.

## Supporting information


**Figure S1:** Control freeze test of overstory species under ambient conditions. Representative bud cross‐sections of (a) Larix laricina and (b) Picea mariana exposed to a series of freeze test temperatures (4°C to −80°C). Images illustrate structural differences and visual browning damage in bud tissues tested in early spring (2 April 2025 for *Larix laricina* and 16 April 2026 for *Picea mariana* ). Red borders and labels indicate temperatures at which visible tissue damage is observed. Box plots show the distribution of cold hardiness values and individual points represent single bud cold hardiness measurements. Mean cold hardiness was −25.1°C for *Larix laricina* and −24.9°C for *Picea mariana*, consistent with the bud browning observed in the dissections for both species.
**Figure S2:** Histogram of measured winter temperatures at (a) 2 m and (b) surface level (0 m) over the course of four winter seasons at the SPRUCE experiment (1 August 2021 to 30 June 2025).
**Figure S3:** Experimental warming reduces snow cover in SPRUCE enclosures. Overhead images taken on the same day (January 17, 2024, ~2:00 PM) showing snow cover across warming treatments at the SPRUCE experiment. Panels show (a) ambient conditions and enclosures maintained at (b) +0.00°C, (c) +2.25°C, (d) +4.50°C, (e) +6.75°C, and (f) +9.00°C above ambient. Increasing warming levels correspond with progressively reduced snow cover within enclosures.
**Figure S4:** Seasonal patterns of air temperature and bud cold hardiness across warming treatments. Seasonal dynamics of air temperature and bud cold hardiness for four species at the SPRUCE experiment from 2021 to 2025. Panels show (a) *Larix laricina* , (b) *Picea mariana* , (c) *Chamaedaphne calyculata* , and (d) *Rhododendron groenlandicum* . Lines represent daily air temperature measured within ambient (blue) and warmed (+9°C; red) treatments, with shaded bands indicating variability across plots. Colored points show measured bud cold hardiness (LTE, °C) across warming treatments (+0.00°C, +2.25°C, +4.50°C, +6.75°C, and +9.00°C above ambient) at each sampling date during the dormant season.
**Figure S5:** Cold hardiness responses to warming relative to ambient conditions. Bud cold hardiness for *Larix laricina* , during 2023–2024. Each panel combines ambient and a single warming treatment.
**Figure S6:** Short‐term thermal responsiveness of cold hardiness across physiological phases and treatments. Relationships between inter‐collection mean air temperature and short‐term changes in bud cold hardiness (∆CH) for (a) overstory and (b) understory species. Negative ∆CH indicates acclimation; positive values indicate deacclimation. Points represent treatment means and ellipses represent 90% confidence regions for each physiological phase: acclimation (golden), midwinter maintenance (dark teal), and deacclimation (sage green).
**Figure S7:** Cumulative intra‐seasonal corrected plot temperature differential (∆*T*, °C) versus target warming treatment (∆*T*, °C). Data shown at two sensor heights: (a) surface level (0 m) and (b) overstory (2 m). Each point represents the mean cumulative intra‐seasonal corrected ∆T for an individual collection and treatment (Ambient, +0.00°C, +2.25°C, +4.50°C, +6.75°C, and +9.00°C). Gray lines connect collection means across treatments within each panel. The dashed line indicates the 1:1 relationship between target and corrected temperatures.
**Figure S8:** Histogram of measured December temperatures for each of four seasons at 2 m at the SPRUCE experiment.
**Figure S9:** Warming sensitivity of bud cold hardiness across seasonal phases. Slopes of warming responses for (a) overstory species and understory (b) species across acclimation (golden), maintenance (dark teal), and deacclimation (sage green) phases. Each point represents the slope of cold hardiness change per °C warming using inter‐collection mean temperatures for an individual collection across four winters. Black curves show the overall seasonal trend; thin gray lines connect points within individual seasons.
**Figure S10:** Bud cross‐sections of understory shrub species across warming treatments. Representative bud cross‐sections of (a) *Chamaedaphne calyculata* (leatherleaf) and (b) *Rhododendron groenlandicum* (Labrador tea) across ecosystem warming treatments. Buds are shown from ambient conditions and warming levels of +0.00°C, +2.25°C, +4.50°C, +6.75°C, and +9.00°C above ambient. Images illustrate structural differences in bud tissues collected in early spring (25 March 2025).
**Figure S11:** Seasonal dynamics of bud cold hardiness in *Larix laricina* across warming treatments. Bud cold hardiness (LTE, °C) measured at multiple sampling dates from 2021 to 2025 at the SPRUCE experiment. Each panel represents a sampling date, with points showing individual plot measurements across ecosystem warming treatments (+0.00°C, +2.25°C, +4.50°C, +6.75°C, and +9.00°C above ambient). Colors indicate warming levels. Solid black lines show linear regression relationships between bud cold hardiness and air temperature across treatments for each sampling date.
**Figure S12:** Seasonal dynamics of bud cold hardiness in *Picea mariana* across warming treatments. Bud cold hardiness (LTE, °C) measured at multiple sampling dates from 2021 to 2025 at the SPRUCE experiment. Each panel represents a sampling date, with points showing individual plot measurements across ecosystem warming treatments (+0.00°C, +2.25°C, +4.50°C, +6.75°C, and +9.00°C above ambient). Colors indicate warming levels. Solid black lines show linear regression relationships between bud cold hardiness and air temperature across treatments for each sampling date.
**Figure S13:** Seasonal dynamics of bud cold hardiness in *Chamaedaphne calyculata* across warming treatments. Bud cold hardiness (LTE, °C) measured at multiple sampling dates from 2021 to 2025 at the SPRUCE experiment. Each panel represents a sampling date, with points showing individual plot measurements across ecosystem warming treatments (+0.00°C, +2.25°C, +4.50°C, +6.75°C, and +9.00°C above ambient). Colors indicate warming levels. Solid black lines show linear regression relationships between bud cold hardiness and air temperature across treatments for each sampling date.
**Figure S14:** Seasonal dynamics of bud cold hardiness in *Rhododendron groenlandicum* across warming treatments. Bud cold hardiness (LTE, °C) measured at multiple sampling dates from 2021 to 2025 at the SPRUCE experiment. Each panel represents a sampling date, with points showing individual plot measurements across ecosystem warming treatments (+0.00°C, +2.25°C, +4.50°C, +6.75°C, and +9.00°C above ambient). Colors indicate warming levels. Solid black lines show linear regression relationships between bud cold hardiness and air temperature across treatments for each sampling date.
**Table S1:** Summary of linear effects model results for cold hardiness across four species. Linear models evaluating the effects of warming treatment (Δ*T*), CO_2_, and collection period on cold hardiness. Values shown are *p* values from sequential ANOVA tests. Adjusted *R* ^2^ represents the proportion of variance explained by fixed effects.

**Table S2:** Sample sizes for low‐temperature exotherm (LTE) measurements. Number of bud cold hardiness (LTE) measurements for each collection, species, and year.

**Table S3:** Comparison of cold hardiness temperature sensitivity among collections. Collection‐specific slopes (β₁) of bud cold hardiness (LTE) regressed on corrected treatment temperature, estimated separately for each species and season, with pairwise comparisons among collections. For each species and season, slopes were compared using estimated marginal trends (emmeans package) with Tukey adjustment (α = 0.05); collections sharing a letter within a season and species did not differ significantly in temperature sensitivity. Slopes for *Larix laricina* and *Picea mariana* are based on 2 m temperature; those for *Chamaedaphne calyculata* and *Rhododendron groenlandicum* on surface‐level (0 m) temperature. These are the slopes shown in Figure 5.

## Data Availability

The raw environmental data and cold hardiness data are available at https://doi.org/10.3334/cdiac/spruce.032 (Hanson et al. 2016) and https://doi.org/10.15485/3408148 (Campos‐Arguedas, Kirchhof, North, Pearson, et al. 2026), respectively. All data and code supporting this study are available through GitHub (https://github.com/apkovaleski/SPRUCE_CHresponses) (Campos‐Arguedas and Kovaleski 2026).
